# S147. ASSOCIATION BETWEEN SOCIAL ANHEDONIA AND TOPOLOGICAL PROFILE OF BRAIN NETWORK IN SCHIZOPHRENIA

**DOI:** 10.1093/schbul/sby018.934

**Published:** 2018-04-01

**Authors:** Zhi Li, Raymond Chan

**Affiliations:** Institute of Psychology, Chinese Academy of Sciences

## Abstract

**Background:**

As a refractory negative symptom, social anhedonia is prevalent in people with schizophrenia spectrum disorders. Furthermore, schizophrenia is conclusively correlated with disorganized functional brain network reflected by topological profile. However, studies on the relationship between social anhedonia and topological properties of functional brain network in schizophrenia were limited to a large extent. In the present study we explored the neurofunctional mechanism of social anhedonia in schizophrenia from the perspective of topological profile of functional brain network.

**Methods:**

Six-minute resting-state fMRI images were acquired from 65 patients with schizophrenia in a 3T SIMENS scanner. Topological properties of functional brain network derived from the resting-state fMRI image, including clustering coefficient, global efficiency and small-worldness were calculated. The social anhedonia of each participant was measured with the Chapman Social Anhedonia Scale. Due to the wide-range of duration of illness in patients with schizophrenia, we included the duration of illness, social anhedonia and their interaction into a generalized linear model to predict the three topological properties, with gender, age and education years as covariates.

**Results:**

We found that the clustering coefficient of brain functional network in schizophrenia increased (p = 0.032, beta = 0.000194 at the minimum network sparsity 35% in which all the nodes were fully connected), whereas the global efficiency decreased (p = 0.005, beta = -0.000022), as the progression of schizophrenia. Although the main effect of social anhedonia in predicting both the clustering efficient and the global efficiency were not significant, its interaction with the duration of illness was significant (p = 0.021, beta = 0.000038 for the clustering coefficient; p = 0.023, beta = -0.000003 for the global efficiency).

**Discussion:**

With the development of schizophrenia, the increase of clustering coefficient and decrease of global efficiency of functional brain network may reflect the pathophysiology of schizophrenia since the onset of illness. Social anhedonia plays as a mediator between the altered topological profile of brain network and the progression of schizophrenia.

